# Assessing central nervous system contributions to accelerate musculoskeletal pain diagnosis and treatment (AsCent): protocol for a mixed-method, prospective observational study

**DOI:** 10.1136/bmjopen-2025-115860

**Published:** 2026-05-18

**Authors:** Georgia Clay, Stevie Vanhegan, Caroline Abbott, Fiona A Pearce, Fiona Moffatt, Kirsty Bannister, Thomas Graven-Nielsen, David A Walsh, Stephanie L Smith

**Affiliations:** 1 Pain Centre Versus Arthritis, University of Nottingham, Nottingham, UK; 2 School of Psychology, Sport Science, and Wellbeing, University of Lincoln, Lincoln, UK; 3 Musculoskeletal, Surgery, Inflammation and Recovery, NIHR Nottingham Biomedical Research Centre, Nottingham, UK; 4 Independent Contributor, University of Nottingham, Nottingham, UK; 5 Independent Contributor, University of Nottingham, St Albans, UK; 6 School of Medicine, University of Nottingham, Nottingham, UK; 7 School of Health Sciences, University of Nottingham, Nottingham, UK; 8 Department of Life Sciences, Imperial College London - South Kensington Campus, London, UK; 9 Center for Neuroplasticity and Pain (CNAP), Department of Health Science and Technology, Aalborg University, Aalborg, Denmark; 10 Rheumatology, Sherwood Forest Hospitals NHS Foundation Trust, Sutton-in-Ashfield, UK

**Keywords:** Musculoskeletal disorders, Back pain, Chronic Pain, RHEUMATOLOGY, PAIN MANAGEMENT

## Abstract

**Introduction:**

Chronic musculoskeletal pain often extends beyond pathology alone. Augmented central pain processing is linked to pain severity, persistence and treatment outcomes. A practical clinical tool is needed to identify individuals likely to have persistent or worsening pain, likely due to augmented central pain mechanisms. Quantitative Sensory Testing (QST) offers mechanistic insight, while the Central Aspects of Pain (CAP) Questionnaire captures symptom profiles that potentially reflect central mechanisms. Combining a brief clinical QST protocol with CAP may support early risk stratification and guide personalised pain management.

**Methods and analysis:**

This prospective observational study will recruit 250 individuals with inflammatory arthritis, osteoarthritis, chronic low back pain or fibromyalgia from existing cohorts, primary or secondary care. Participants will complete validated patient-reported outcomes at baseline, 6 and 12 weeks, with no additional intervention. The risk stratification tool completed at baseline will include clinical QST (Pressure Pain Threshold, Temporal Summation of Pain, Conditioned Pain Modulation), tender point count and the CAP questionnaire. Baseline laboratory versions of the clinical QST, plus Heat Pain Threshold, Offset Analgesia and the Central Sensitisation Inventory short form-9 questionnaire, will provide pain profiling to evaluate the predictive validity and psychometric properties of the tool. Data collection will include demographics, medical history, cognitive and neurological assessments and sleep quality via actigraphy (Actigraph wGT3X-BT). Interviews with patients and healthcare professionals will inform refinement, feasibility and acceptability of the tool.

**Ethics and dissemination:**

Ethical approval was obtained from the Yorkshire & The Humber—South Yorkshire Research Ethics Committee (reference number: 24/YH/1062). Findings will be disseminated through peer-reviewed publications, conference presentations and patient-facing summaries and podcasts. The study aims to develop a clinically feasible tool to identify individuals at risk of persistent or worsening pain due to augmented central pain processing, enabling targeted treatment strategies.

**Trial registration number:**

NCT06518278.

**STRENGTHS AND LIMITATIONS OF THIS STUDY**

- The proposed tool integrates mechanistic insight (Quantitative Sensory Testing (QST)) with self-reported symptom profiles to offer a comprehensive assessment of augmented pain processing.
- The clinical QST protocol mimics the laboratory measures using low-cost equipment already available within the National Health Service in the UK, alongside the short questionnaire, designed to promote implementation.
- Recruiting individuals with a range of musculoskeletal conditions enhances generalisability across common pain presentations.
- Maladaptive pain processing is multidimensional where an individual may demonstrate hypersensitivity to one or multiple QST modalities; while core modalities have been included, the full armamentarium could not be performed for practical reasons.

## Introduction

 Pain is a primary symptom of musculoskeletal conditions, affecting 1.7 billion people worldwide.^1^ The International Association for the Study of Pain (IASP) defines pain as an unpleasant sensory and emotional experience associated with or resembling that associated with actual or potential tissue damage.^2^ Chronic pain is categorised based on activation of nociceptors due to actual or threatened damage (nociceptive pain) or lesions or disease affecting the somatosensory nervous system (neuropathic pain),^2^ but these alone often fail to explain chronic musculoskeletal pain.^3^

Musculoskeletal pain manifests as localised, regional or widespread pain.^4^ Peripherally augmented pain is typically localised but may also extend to adjacent regions and be referred. Centrally augmented pain is often widespread, multifocal, fluctuating in location and intensity, and accompanied by comorbidities also found with localised pain: fatigue, sleep and mood disturbances, cognitive impairment and hypersensitivity.^5^

Chronic pain is associated with altered activation, modulation and connectivity of nociceptive pathways in the central nervous system (CNS).^5^ Quantitative Sensory Testing (QST) has demonstrated the presence of localised and spreading pain hypersensitivity in chronic pain conditions.^46^,^12^ Decreased pressure pain threshold (PPT) at the site and away from the site of tissue injury (eg, an arthritic joint) is believed to demonstrate altered CNS processing of afferent nociceptive signals.^9^ Increasing pain during Temporal Summation of Pain (TSP) assessed by repeated painful stimuli^13^ has been attributed to spinal cord facilitation of nociceptive signalling.^14^ Conditioned Pain Modulation (CPM) reflects a proxy measure of efficiency in descending modulatory controls.^15^ Offset analgesia reflects spinal temporal filtering of nociceptive signals.^16^ The CNS mechanisms underlying Offset Analgesia differ from those underlying a CPM effect.^17^ In both cases, the cost, time requirements and reliance on specialised equipment and expertise make it impractical for routine clinical use encompassing these tests. Clinical adaptations of QST, using alternative equipment or approaches, hereafter referred to as Clinical QST, have been developed for neuropathic pain.^18^,^29^ A similar approach could help identify individuals whose pain is likely driven by augmented central pain pathways.

Although QST evaluates augmented central pain processing, it may not fully capture higher-order dysfunction affecting emotion, cognition, sleep and fatigue driven by affective states. Self-report questionnaires may better assess and capture these aspects, with studies showing associations between questionnaire responses and QST outcomes.^30^,^34^ The Central Aspects of Pain (CAP) questionnaire, initially developed for knee pain^35^ and later refined for other musculoskeletal conditions,^36 37^ includes items associated with neuropathic-like pain, fatigue, anxiety, depression, catastrophising, sleep, cognitive impact and pain distribution.^35 38 39^ CAP forms a unitary construct more strongly associated with QST than any individual characteristic alone.^38^ Similarly, the Central Sensitisation Index short form (CSI-9) was developed from medically unexplained symptoms associated with pain not adequately explained by tissue damage.^40^,^44^ While practical, questionnaires lack the mechanistic precision to assess augmented pain pathways indirectly.

We propose integrating clinical QST with questionnaires to develop a risk stratification tool to identify individuals at risk of persistent or worsening pain likely due to augmented central pain processing. This tool is hypothesised to be feasible, reliable and practical for clinical settings, enabling timely and targeted pain management. Its goals are to:

1. Identify individuals at risk of persistent or worsening pain likely due to augmented central pain processing.
2. Identify those likely to benefit from CNS-targeted interventions.
3. Detect early treatment responses even before symptom improvement.

A targeted approach would move beyond ‘one-size-fits-all’, trial and error approach, reduce unnecessary treatments,^45 46^ improving confidence in medical care, fostering realistic expectations^47^ and potentially healthcare economic benefits. This study co-developed the tool with individuals experiencing chronic musculoskeletal pain, ensuring its relevance, feasibility and impact.

### Objectives

The primary objective is to:

1. Assess the feasibility, acceptability and reliability of the CAP questionnaire and clinical QST to identify people at risk of persistent and/or worsening pain arising or augmented by augmented central pain processing across a range of musculoskeletal pain conditions.
2. Determine the ability of the clinical QST and CAP questionnaire, alone or combined, to predict persistent and/or worsening pain at 6 and 12 weeks.
3. Determine the ability of clinical QST and CAP alone or combined with other proxy measures of augmented central pain processing, demographics and clinical variables to improve prediction of persistent and/or worsening pain at 12 weeks.
4. Validate clinical QST and CAP in relation to laboratory QST as an outcome measure for augmented central pain processing.
5. Evaluate associations between the risk tool (clinical QST and CAP) and key patient-centred outcomes linked to augmented central pain processing.

The secondary objectives are to:

1. Determine the ability of the clinical QST and CAP questionnaire, alone or combined, to predict persistent and/or worsening pain at 6 and 12 weeks.
2. Determine the ability of clinical QST and CAP alone or combined with other proxy measures of augmented central pain processing, demographics and clinical variables to improve prediction of persistent and/or worsening pain at 12 weeks.
3. Validate clinical QST and CAP in relation to laboratory QST as an outcome measure for augmented central pain processing.
4. Evaluate associations between the risk tool (clinical QST and CAP) and key patient-centred outcomes linked to augmented central pain processing.

## Methods and analysis

### Study design and setting

This multisite, prospective observational study will recruit adults with inflammatory arthritis, osteoarthritis, fibromyalgia or chronic low back pain. The study design follows the recommendations of the Consolidated Standards of Reporting Trials (CONSORT) 2010 extension for observational studies.^48^ It is reported following the Strengthening and Reporting of Observational Studies in Epidemiology guidelines (online supplemental material 1).^49^ The study started recruitment in October 2024, is currently recruiting and is expected to end in April 2027.

### Patient and public involvement

Patient and public involvement (PPI) follows the National Institute for Health and Care Research UK Standards for Public Involvement framework. Individuals with lived experience of musculoskeletal pain were pivotal in the development of this study. The study was co-designed through online discussions (n=9) and peer reviews with individuals experiencing musculoskeletal pain, influencing decisions on the research theme, assessment measurement tools and the participant journey. The CAP questionnaire was initially developed and later refined with patient and public contributors.^35 36 38 39^

To enhance accessibility and engagement, study materials were co-created, and the participant journey was refined through discussions and walkthroughs of the study visits. Their feedback ensured that materials were clear, relevant and patient-centred.

People with musculoskeletal pain are active members of this paper’s study steering committee and co-authors (SV and CA). They contribute throughout the study by attending steering group meetings, ensuring lived experience perspectives inform decision-making, supporting study materials and protocol development, refining participant-facing documents and interpreting and disseminating study findings to ensure findings are meaningfully interpreted and communicated from a patient perspective.

SV and CA were coapplicants on the successful funding applications approved by Arthritis UK and the European Alliance of Associations for Rheumatology (EULAR), who integrated PPI in the review process. This embedded PPI approach ensures that the study remains relevant, feasible and reflective of lived experience, maximising its impact.

### Participants

Inclusion (must meet all criteria):

- Aged 18 years or older.
- One or more of the following physician confirmed or self-reported diagnoses: fibromyalgia, osteoarthritis, chronic low back pain, inflammatory musculoskeletal conditions (eg, rheumatoid arthritis (RA), psoriatic arthritis (PsA), axial spondylarthritis).
- Musculoskeletal diagnosis and pain onset ≥3 months.
- Self-reported pain ≥3/10, where 0 is ‘no pain’ and 10 is the ‘worst pain imaginable’ for most days in the 3 months before baseline.

Exclusion (if any of the following apply):

- Unable to give informed consent.
- Terminal or uncontrolled medical or mental health condition precluding them from completing the study protocol or pose a significant risk to themselves or staff.
- Unable to comprehend spoken to written English, due to not all questionnaires being translated and validated in other languages, and due to the nature of QST, individuals need to fully comprehend the testing procedure to ensure reported outcomes are valid.

Participants will be identified through primary or secondary healthcare services, including Sherwood Forest NHS Foundation Trust, Primary Integrated Community Services, the Investigating Musculoskeletal Health and Wellbeing research cohort^50^ and Central Aspects of Pain in Rheumatoid Arthritis Cohort.^37^ Individuals recruited from primary or secondary care will have a diagnosis done by a physician of one or more of the above conditions. Individuals recruited from the community or research cohorts will have a self-reported diagnosis. Individuals meeting the above criteria will be invited to participate in the study and provided with an invitation pack containing an invitation letter, a participant information sheet, a reply slip and a pre-paid envelope, or a link to access all of these and a form to fill in. Participants will undergo a telephone screening to confirm eligibility, and an appointment will be scheduled at a mutually convenient time. At the study appointment, participants will be screened a final time before providing written informed consent and undertaking study assessments. Recruitment will ensure the study populations represent the four diagnostic groups and display age, sex, social and ethnic diversity representative of the study population. Purposive sampling will be used to ensure that less common conditions, such as inflammatory arthritis, have sufficient power to allow proper analysis. Given the prevalence of multiple long-term musculoskeletal conditions, we anticipate that this approach will yield a representative chronic pain population. All individuals will provide written informed consent prior to participating in the study (see online supplemental material 2). Reasonable travel expenses will be covered. Additional incentives will not be provided.

### Outcomes

Primary outcomes:

1. The risk for persistent pain or pain worsening to a clinically important extent, using the risk stratification tool (combined clinical QST and CAP).
2. Sensitivity and specificity of the risk stratification tool to identify people at risk of persistent and/or worsening pain.

Secondary outcomes:

1. External validity of the risk stratification tool against laboratory QST and validated questionnaires addressing central sensitisation-related characteristics.
2. Internal validity of the risk stratification tool.
3. Pain prognosis: Prediction of pain at 12 weeks by the risk stratification tool, CAP and clinical QST separately.
4. Mechanistic underpinning: Investigate, according to the assessments outlined, which pain mechanisms contribute to risk levels identified using the risk stratification tool.
5. Acceptability and feasibility of the risk stratification tool by patients, healthcare professionals (HCPs) and researchers within both research and clinical environments.
6. Refinement of the risk stratification tool by patients, HCPs and researchers.
7. Inter-rater and intrarater reliability of the risk stratification tool.

### Experimental procedures

**Figure 1:**
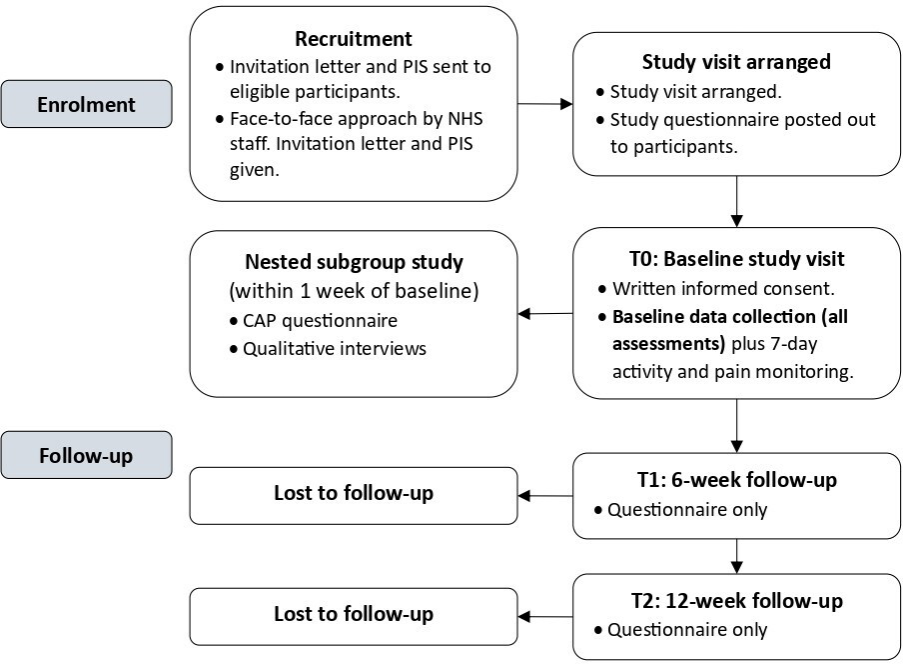
CONSORT (Consolidated Standards of Reporting Trials) participant flow diagram. CAP, Central Aspects of Pain; NHS, National Health Service; PIS, Participant Information Sheet; T0, time 0 (baseline); T1, time 1 (6 weeks); T2, time 2 (12 weeks).

Participants will attend a single study visit that includes the new tool, patient-reported outcome measures, laboratory QST, and clinical assessments. They will be asked to wear an activity monitor for 1 week to evaluate the impact of sleep on pain. Pain will also be reported via SMS text messages weekly for 12 weeks. A subgroup of individuals will complete an additional CAP questionnaire and qualitative semistructured interviews within 1 week of their baseline visit. Patient-reported outcome measures (questionnaire booklet) will be repeated at 6 and 12 weeks. The CONSORT flow diagram (figure 1) outlines the participants’ journey. Participants may withdraw at any time without providing a reason, without prejudice to their medical care.

The combined tool will include the CAP questionnaire and clinical QST modalities, replicating laboratory QST using low-cost equipment currently available within the UK National Health Service (NHS) (table 1). The CAP questionnaire^35 36 38 39^ has been selected because it was developed based on the current mechanistic understanding of augmented central pain processing and the potential patient-reported effects. CAP includes eight items strongly associated with QST in musculoskeletal pain.^38^ It displays excellent psychometric properties, is feasible, patient-acceptable and can predict pain prognosis in musculoskeletal conditions so far investigated.^35^,^3751^

**Table 1:** Laboratory versus clinical QST assessments for pain hypersensitivity

Modality	Laboratory	Clinical
**Pressure Pain Detection Threshold (PPT**) **Protocol:** Pressure is increased at 50 kPa/s until the sensation of pressure changes to pain^53^ **Test sites:** non-dominant brachioradialis	**Equipment:** Pressure algometer (Algomed, Medoc) 1 cm^2^ tip	**Equipment:** Handheld dynamometer (Wagner Force Ten) 1 cm^2^ tip
**Temporal Summation of Pain** **Protocol:** apply single stimuli, apply 10 stimulations at 1/s and rate pain/sharpness after 1 and 10 **Test sites:** dominant brachioradialis	**Equipment:** Pinprick stimulator (256 mN pinprick)	**Equipment:** Von Frey Filament (256 mN)
**Conditioned Pain Modulation** **Protocol:** PPT is performed preconditioning and postconditioning. Conditioning: A blood pressure cuff will be inflated until the distal radial pulse cannot be palpated. Participants are then asked to squeeze a ball until pain reaches 4/10. **Test sites:** non-dominant brachioradialis	**Equipment:** Blood pressure cuff, pressure algometer (Algomed, Medoc)	**Equipment:** Blood pressure cuff, handheld dynamometer 1 cm^2^ tip (Wagner Force Ten)

QST, quantitative sensory testing.

**Figure 2:**
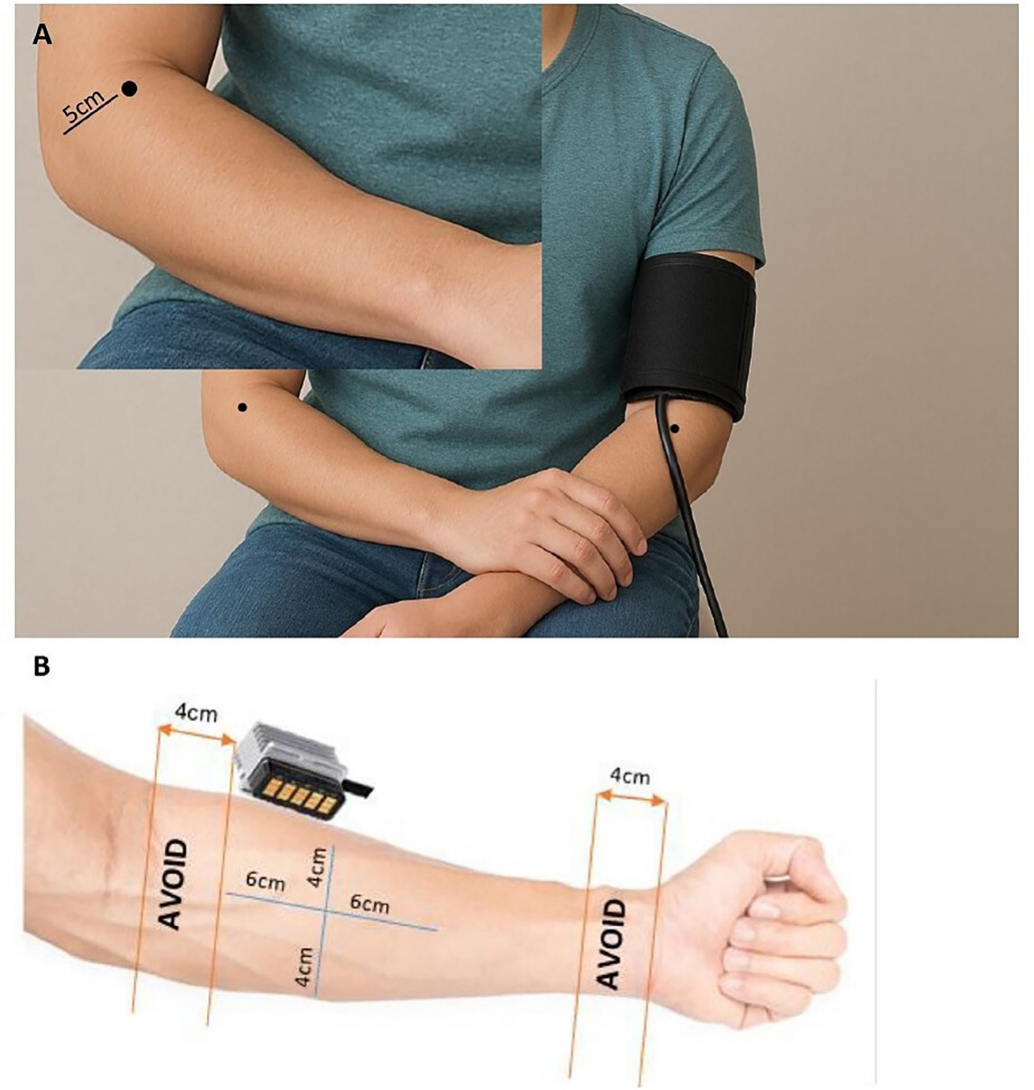
**(**A) Pressure Pain Detection Threshold, Temporal Summation of Pain and Conditioned Pain Modulation test location, 5 cm from the lateral epicondyle on the brachioradialis muscle. (B) Heat Pain Threshold and Offset Analgesia probe location on the volar forearm, 4 cm distal to the elbow joint and 4 cm proximal to the wrist using the centre line from the elbow to the wrist, a cross is marked out 6 cm deep and 4 cm wide.

QST will be assessed on the brachioradialis muscle belly 5 cm from the lateral epicondyle^52^ of the dominant (PPT, CPM) and non-dominant arm (TSP), and the volar forearm of the dominant arm (Heat Pain Threshold (HPT), Offset-analgesia) (figure 2). Each laboratory and clinical QST protocol test will be undertaken in a standardised order (PPT, TSP, CPM, HPT, then offset analgesia). The order of laboratory and clinical QST will be randomised to minimise the potential for sequential effects to confound comparisons between the two modalities. These measures have previously been well tolerated by individuals with musculoskeletal pain and will be conducted regardless of their pain status.

Handheld pressure algometer (Algomed, Medoc, Israel (laboratory), Force Ten FDX, Wagner Instruments, USA (Clinical)) with a 1 cm^2^ tip will be used to apply pressure at a gradual rate of 50 kPa/s^53^ to assess PPT. The participant will press a handheld button (laboratory) or say ‘Stop’ (Clinical) when the sensation of pressure changes to pain or discomfort. PPT will be calculated as the mean of three replicates separated by at least 1 min or until any lingering feelings have subsided. Lower values indicate greater sensitivity.

A 256 mN punctate stimulator (Laboratory—MRC Systems, Germany) or monofilament (Clinical—OptiHair2, MRC Systems, Germany) will assess TSP. A single stimulus will be applied, followed by a train of 10 repeated stimuli at a rate of 1/s^54^ based on prior training. The participant will be asked to rate their pain intensity from 0 ‘no pain or sharpness’ to 10 ‘worse pain or sharpness’ on a Visual Analogue Scale (VAS) after the single and the average of 10 repeated stimuli. TSP will be calculated as the VAS of 10 stimuli minus the VAS of a single stimulus.^51^ An average of 10 will be used to include the most painful stimuli, as they don’t always occur as the 10th stimulus.^55^ The mean of two replicates separated by 5 min will be calculated.^51^ Large positive values for TSP indicate greater sensitivity (likely more efficient spinal integration).

Using the Advanced Pain Discovery Platform CPM protocol, PPT will be measured before and after a pain-conditioning stimulus. The unconditioned stimulus will be the average of three trials on the non-dominant brachioradialis muscle. A 15 cm wide pressure cuff on the dominant forearm will be inflated to occlude arterial blood flow. Participants will be asked to squeeze a stress ball until they indicate pain levels of 4/10 (0=no pain, 10=worst pain imaginable) due to ischaemic pain,^56^ at which point PPT is performed again before the cuff is released. The CPM effect will be calculated as the mean of three unconditioned PPT minus the conditioned PPT.^56^ Thus, negative values indicate an inhibitory CPM effect and positive values a facilitatory effect.^56^ To account for measurement error, the within-participant coefficient of variation (CoV) of the unconditioned PPT will be determined; only the CPM effect greater in magnitude than the CoV will be considered a true response.^57^

HPT will be assessed using a 6.6×4×4.4 cm probe (TCS II, QST.Labs, France) applied to the non-dominant volar forearm. To overcome adaptation or sensitisation, the forearm is divided into four zones (figure 2), with the probe moved from zone to zone between stimuli.^58^ The method of limits will be used to establish the heat thresholds. A computer-controlled heat stimulus will be applied, with the baseline temperature set at 32°C and increasing at a rate of 1°C/s to a maximum of 50°C.^57^ The participant will indicate using a handheld button when the sensation of heat changes to one of pain, at which point the test is stopped. HPT is calculated as the mean of three replicates taken 10 s apart. Lower HPT values indicate greater sensitivity to thermal pain.

Offset analgesia will be measured using a tonic heat stimulus train to the skin of the non-dominant volar forearm (figure 2). The study temperature will be individualised. Starting at baseline (32°C), the first temperature will be set to HPT for 10 s, then returned to baseline and increased by 1°C. The participant will continuously rate their pain on an electronic VAS (eVAS). When the eVAS reaches 50/100, the test will be stopped^58^ and the actual temperature recorded. Pain^50^ will be determined as the average of two trials separated by 1 min. To ensure the tonic heat stimulus is noxious for the offset analgesia protocol, the following rule will apply:

Pain^50^<45°C then tonic heat stimulus=Pain^50^+2°C.

Pain^50^≥45 °C then tonic heat stimulus=Pain.^50^

The offset analgesia paradigm will consist of three continuous phases: (T1) initial tonic heat stimulus (5 s); (T2) tonic heat stimulus+1 °C (5 s); (T3) tonic heat stimulus (20 s).^16^ After T3, the temperature will decrease back to baseline (75°C/s). The control paradigm will consist of a constant tonic heat stimulus for 30 s. Participants will continuously rate their pain from 0 as ‘no pain’ to 100 as ‘worst pain imaginable’ on the eVAS.^10^ Two offset analgesia and two control trials will be undertaken in random order, randomised to the volar forearm zone. Offset analgesia will be determined based on the following approaches: Peak eVAS (T2); min eVAS (T3); ΔeVAS=peak eVAS−min eVAS; ΔeVAS corrected = (ΔeVAS/peak eVAS)×100.^59^ The following approaches will also be explored: difference in average pain rating T3 (16 s–20 s) between control and offset analgesia,^60^ difference in average pain rating T1 (4 s–5 s) and T3 (16 s–20 s). If the alternative approaches are more reliable in this population, the most reliable will be taken forward. Positive scores indicate the presence of offset analgesia.

In addition to the clinical QST, tenderness to nail blanch pressure of the assessor (approx. 4 kg) will be assessed at 18 sites (bilateral occiput, trapezius, supraspinatus, low cervical, second rib, lateral epicondyle, gluteal, greater trochanter, knee). Each site is classified as ‘tender’ or ‘not tender’, according to whether the participant felt the stimulus as only pressure, or as pain.^61^ The total number of tender sites will be used to index pressure pain sensitivity.

Immediate and delayed word recall (memory), animal naming (semantic fluency) and letter cancellation (visuospatial) will assess cognitive function.^62 63^ Neuropathy will be evaluated as a potential confounder through sensory and reflex neurological assessment, including knee and ankle reflexes, along with four sensory tests (touch pressure, pinprick, vibration, joint position) on the dorsal surface of the great toe using the modified Neuropathy Impairment Score in the Lower Limb.^64^

Sleep quality will be assessed using the self-reported Pittsburgh Sleep Quality Index^65^ and indirectly through wrist-worn actigraphy (wGT3X-BT, Actigraph, USA) on seven consecutive nights following the baseline study visit. Sleep diaries will validate actigraphy data, before calculating sleep onset latency, total sleep time, number of awakenings, total wake time and sleep efficiency.^66^

All participants will complete the Widespread Pain Index and the Symptom Severity Scale (SSS) to classify fibromyalgia according to the American College of Rheumatology (ACR) fibromyalgia criteria.^67^ Possible or probable nociplastic pain will be classified using the IASP Nociplastic Pain clinical criteria.^68^ Hand, hip, and knee osteoarthritis will be classified using the EULAR-ACR clinical criteria.^69^,^72^ For individuals reporting inflammatory arthritis, participants will be assessed against condition-specific criteria: ACR/EULAR rheumatoid arthritis (RA)^73^; the Modified New York criteria for axial spondyloarthritis^74^; the Classification Criteria for Psoriatic Arthritis^75^ for PsA. Individuals with RA and PsA will be assessed using the 28-joint Disease Activity Score.^76^

All participants will have a baseline blood sample taken. High-sensitivity C reactive protein will be evaluated as an inflammatory marker.

Questionnaires completed at baseline, 6, and 12 weeks will assess clinical characteristics associated with maladaptive pain processing, patient-centred outcomes and potential confounders. Characteristics related to augmented central pain processing will be evaluated using the Cognitive Failures Questionnaire,^77^ Hospital Anxiety and Depression Scale,^78^ Bristol Rheumatoid Arthritis Fatigue Scale,^79^ Pain Catastrophising Scale,^80^ Self-Reported Leeds Assessment of Neuropathic Signs and Symptoms^81^ and the Pittsburgh Sleep Quality Index.^65^ The CSI Short-Form (CSI-9)^40^,^43^ will be used as an alternative for comparison with CAP.

Patient-centred outcomes will assess pain severity (Numerical Rating Scale^82^), quality (McGill^83^) and periodicity (pain diaries^84 85^), disability (Health Assessment Questionnaire^86^) and global quality of life (36-item Short Form Survey^87^). Potential confounders will also be included in the questionnaire, including demographics (age, sex, ethnicity, social deprivation,^88^ amenorrhea status), comorbidities (Rheumatic Disease Comorbidity Index^89^). Regular and current medication used will be classified as analgesic (subclassified: paracetamol, Non-steroidal anti-inflammatory drugs, anti-neuropathic (antidepressant, anti-convulsive), strong/weak opioid, other).

### Stopping criteria

Participants will be informed that they can withdraw their consent at any time during the trial without affecting their healthcare and without needing to give a reason. If participants decide to withdraw, the primary reason for discontinuation will be determined and recorded where possible. Individuals will be informed that, should they withdraw, the data collected to that point cannot be erased and may still be used in final analyses.

### Nested qualitative interviews

Semistructured interviews will be conducted with patients, HCPs (eg, consultants, physiotherapists, nurses, occupational therapists), and researchers to explore their perceptions of the proposed stratification tool. Findings from the interviews will determine the tool’s acceptability and feasibility and inform any necessary refinements to the tool’s procedure based on participants’, HCPs’ and researchers’ experiences. The interview schedules for participants, HCPs and researchers (onlinesupplemental materials 3  4) will be informed by the Extended Normalisation Process Theory^90 91^ and our public contributors to develop an understanding of how the proposed tool may be implemented within healthcare practices. All interviews will be audio-recorded with consent via Microsoft Teams or a Dictaphone, either online, face-to-face or by telephone, based on the individual’s preference. Recordings will be transcribed verbatim, and any identifiable information will be removed.

Participants will be purposively sampled initially, with theoretical sampling later to explore emerging themes. Approximately 27 participants, 22 HCPs and researchers will ensure that perspectives across all conditions—gender, age, ethnicity, social deprivation, job role and career level—are represented (table 2). Participants will be individuals who have experienced the new tool as part of the study. HCPs and researchers will be recruited via Cwm Taf Morgannwg University Health Board, Sherwood Forest NHS Foundation Trust, Primary Integrated Community Services, social media, word of mouth and advertising through the British Pain Society. All HCPs and researchers will be provided with an invitation letter, participant information sheet and e-consent (online supplemental material 5) to complete if interested. Topics may include the proposed utility and feasibility of the assessments for the tool, including challenges and barriers and how it may fit into clinical practice, patient burden, any unexpected or undesirable side effects experienced, attitudes and behaviours towards the tool, overall experience and any previous experience with pain assessment tools. The development of interview questions may be iterative and informed by early interviews, with later interviews incorporating additional questions and each round exploring themes from previous rounds in greater depth. One-to-one interviews will be conducted by researchers with appropriate experience in qualitative research.

**Table 2:** Sampling framework and the number of participants for each characteristic

Participants	Subpopulations	What we need	Sample	Total sample
Patients with musculoskeletal conditions	Osteoarthritis	Male: Female 2:4 Age (40–65, >65 years) Socioeconomic+ethnic variation	6	27
	Inflammatory arthritis	RA, PSA, axSpA, 3:3:3 Male: Female 1:2 (RA), 1:1 (PSA), 2:1 axSpA Age (18–39, 40–65, >65 years) Socioeconomic+ethnic variation	9	
	Fibromyalgia	Male: Female 2:4 Age (18–39, 40–65, >65 years) Socioeconomic+ethnic variation	6	
	Chronic low back pain	Male: Female 2:4 Age (18–39, 40–65, >65 years) Socioeconomic+ethnic variation	6	
Researcher	Early career researcher		2	4
	Metrologist/research nurse		2	
Healthcare professionals	Pharmacist	Community vs NHS	2	18
	Physiotherapist	Pain specialist vs no specialist Early vs established	4	
	Occupational therapist	Pain specialist vs no specialist Early vs established	4	
	Nurse	Pain specialist vs no specialist Early vs established	4	
	Rheumatologist/consultant	Early vs established	2	
	General practitioner	Early vs established	2	

axSpA, axial spondylarthritis; NHS, National Health Service; PSA, psoriatic arthritis; RA, rheumatoid arthritis.

Interviews will take place within 1 week of participants’ experience with the stratification tool to ensure accurate recall. They may be conducted face-to-face in a private room, by phone or online via Microsoft Teams, at the interviewee’s discretion. For HCPs, researchers and participants requiring follow-up interviews, short videos of the stratification tool will be provided before the interview. The HCPs and researchers will be recruited through primary and secondary care, via social media and through connections with relevant gatekeepers and the research team.

### Data management

The collection, storage, processing and disclosure of personal information, along with data management, will comply with the requirements of the General Data Protection Regulation 2018. Data handling will adhere to the Sponsor (University of Nottingham) and study sites’ policies and procedures. All personal data will be anonymised, and any further data analysis will be conducted without reference to personally identifiable information. The sponsor will oversee the conduct of the trial.

### Sample size

A sample size of 250 participants at baseline, with an anticipated 200 individuals completing the follow-up questionnaires, accounting for 20% attrition.^92^ Provides additional statistical power and robustness to detect differences, allowing for more precise estimates of interactions and better management of potential missing data, therefore enhancing the validity of our findings.

Rasch measurement theory (RMT) to determine the structural validity of the risk stratification tool assumes that item calibrations lie within ±½ logit of stable values within a 99% CI, resulting in an overall sample size between 108 and 243, from best to poor targeting.^93 94^ This aligns with the Consensus-based Standards for the selection of health Measurement Instruments recommendations RMT analysis.^95^

Pain profiles will be identified irrespective of condition, using multivariate models with 5 covariates, 95% power and an effect size of 0.5 would require 236 individuals to complete baseline assessments.^51^ For longitudinal modelling, assuming a correlation coefficient of 0.15 with 12-week pain, 164 participants with α=0.05 and 95% power.

Interviews will be conducted until theoretical saturation, the point at which additional data do not reveal any new themes that achieve conceptual depth.^96^ It is anticipated that 27 interviews will be conducted with participants and 22 HCPs and researchers (table 2).

### Data analysis

1. Pain profiling will be undertaken irrespective of condition. Multivariate modelling (principal component analysis, cluster analysis or latent class analysis) to identify distinct pain patterns.
  - Between-group comparisons (eg, independent T-tests, Mann-Whitney U) will explore between pain profile comparisons.
  - Associations between pain profiles and clinical outcomes (eg, pain intensity, pain-related worrying, disability, quality of life) will be examined.
  - Sensitivity analysis will be conducted between diagnostic groups and between matched samples.
2. We believe that QST will identify somatosensory pain profiles, while CAP will identify psychological hypervigilance pain profiles, each associated with augmented central pain processing. Using the pain profiles combined with pain at 12 weeks, we will identify any relationship between potential augmented central pain processing and pain prognosis at 12 weeks (eg, persistent/worsening pain, pain>patient acceptable symptom state), informed by patterns of pain over 12 weeks and knowledge.
3. Pain prognosis: Bivariable correlations will identify and permit comparison between baseline factors significantly associated with 3-month pain prognosis. Separate multivariable models will determine whether the CAP questionnaire, clinical QST or laboratory QST can increase prognostic precision beyond other clinical and demographic variables significantly associated with pain prognosis. Multivariable modelling will define whether and which combination of the CAP questionnaire and QST importantly improves prognostic determination. The proportion of the prognosis explained by each and by both the CAP questionnaire and clinical or laboratory QST will be determined.
4. The reliability and validity of the stratification tool (combined CAP and clinical QST) will be tested across the study population, with sensitivity analysis conducted across the diagnostic groups, age, sex, social and ethnic strata.
  - Test–retest reliability will be assessed using intraclass correlation between repeated CAP questionnaires and between two raters for simplified clinical QST.
  - RMT, Cronbach’s alpha and confirmatory factor analysis will evaluate internal validity, consistency and factor structure. If multiple constructs with significant residual values exist, this will be further explored using principal component analysis of residuals, the eigenvalue criterion, residual correlations and theoretical understanding.
  - Correlation analysis will assess external validity against self-reported questionnaires and laboratory QST.
5. Mechanistic underpinning: The dataset will be randomly divided into development and validation sets. Exploratory and confirmatory factor analyses will determine whether there are subgroups of the population for whom the stratification tools function differently. Correlations between CAP scores and clinical and laboratory QST indices of augmented central pain processing will be identified. Bivariate and multivariable models will ascertain whether associations may be explained by one or more characteristics linked to maladaptive pain processing (sleep disturbance, fatigue, anxiety, depression, cognitive impact, catastrophising).
6. Relevance to patient-centred outcomes: Bivariate associations between CAP or QST and patient-reported outcome measures scores will be determined through linear regression. The proportion of participant-reported pain or other patient-reported outcome measures explained by CAP scores, clinical and/or laboratory QST measures, will be identified as R^2^ in multivariable regression.
7. Qualitative data will be analysed using reflexive thematic analysis following the stages outlined by Braun and Clarke.^97^ A reflexive journal will be kept throughout the data collection process. Data will be coded inductively primarily, while also being informed by the Normalisation Process Theory^90 91^ commonly used to support evaluation in health interventions and inform the tool’s theoretical acceptability and feasibility. A ‘critical friend’^98^ will code a sample of transcripts independently until consensus is reached. After the consensus is reached, all remaining transcripts will be coded. NVivo V.15 will assist in managing, analysing, and visualising the qualitative data set. A thematic map may also visually represent the main themes developed.

## Ethics and dissemination

The University of Nottingham sponsors the study (Sponsor reference 24030). Ethical approval was granted by the Yorkshire & The Humber—South Yorkshire Research Ethics Committee, reference number: 24/YH/1062, protocol version 1.0, 13 Jun 2024. The trial was prospectively registered on 23 July 2024 (ClinicalTrials.gov NCT06518278).

The protocol and results will be submitted for publication in open-access, peer-reviewed scientific journals and presented at leading international and national conferences, including the Arthritis UK Pain Centre scientific and educational meetings, to inform research and clinical practice. Lay summaries of the findings will be developed in partnership with our lived experience research partners and the Arthritis UK Pain Centre Patient and Public Advisory Group. The findings will also be posted on the established Arthritis UK Pain Centre website and presented at annual stakeholder engagement events. We will collaborate with the Communications Teams at the University of Nottingham and Arthritis UK to disseminate findings through media releases and public engagement activities.

## Discussion

The clinical evaluation of how augmented central pain processing contributes to musculoskeletal pain currently needs to be standardised and undertaken more frequently to help determine more effective treatments. There is an urgent need for robust, standardised assessment. This study combines a simple self-reported questionnaire (CAP) with clinical QST to optimally identify those at risk of poor pain outcomes likely due to augmented central pain processing. Such a tool would be valuable in clinical settings, where it could identify individuals who may benefit from specific, targeted treatments. Follow-up interviews with participants, HCPs and researchers will further our understanding of the feasibility and usefulness of the proposed risk stratification tool in clinical and research settings and aim to reduce barriers to implementation.

Various augmented central pain processes arise at different times and intensities within an individual. QST and questionnaires are intended to capture psychophysical and higher-order pain processing, respectively, each accounting for a small proportion of the other’s variance. Combining these tools, rather than using them in isolation, may offer a clearer understanding of the likely mechanistic underpinning of augmented central pain processing and improve the ability to identify individuals at risk of poor outcomes likely due to augmented central pain processing. By integrating QST protocols, which mimic laboratory measures and utilising low-cost equipment currently available in healthcare settings, plus combining the quantitative data with interviews to assess the tools’ acceptability, usability and real-world applicability, we aim to ensure implementation in clinical practice. If implemented in clinical practice, the risk stratification tool could assist in diagnosing, measuring, and evaluating treatment for individuals with a range of chronically painful musculoskeletal conditions.

The risk stratification tool will undergo rigorous psychometric testing to establish its validity in predicting the risk of persistent or worsening pain, likely due to augmented central pain processing, in individuals with inflammatory arthritis, osteoarthritis, chronic low back pain, or fibromyalgia. Recruiting across a range of musculoskeletal conditions enhances the generalisability across common pain conditions. We anticipate that a single tool may be valid across chronic musculoskeletal conditions, based on the premise that different musculoskeletal conditions share the same augmented central pain processes and pain prognosis.^5^ Sensitivity analysis will determine whether the risk stratification tool behaves the same, regardless of the condition(s). We will assess the predictive capabilities of the risk stratification tool individually and determine whether the combined stratification tool is more effective than CAP, clinical or laboratory QST.

The current study uses QST modalities to assess augmented central pain processing in musculoskeletal pain. The qualitative data will refine the risk stratification tool to ensure it is feasible and acceptable for clinical practice within the patient population. We will examine the current barriers to assessing and measuring maladaptive pain processing in clinical practice. This mixed-methods approach will enable us to develop a risk-stratification tool that is likely to be more easily implemented in clinical practice. The risk stratification tool will also facilitate the evaluation of novel and existing treatments for efficacy in homogeneous clinical populations at the highest risk of poor pain outcomes.

Augmented central pain processing occurs in a multidimensional manner. We have selected laboratory QST measures that have been published and validated to serve as proxy measures of pain processing. For each clinical measure, we have adhered to the standardised laboratory protocol, employing low-cost equipment currently available in clinical practice. While we have incorporated as many QST modalities as possible, some, such as vibration or thermal grid illusion, have not been included due to a lack of equipment or the development of new techniques during the study. This suggests that, as equipment and measures continue to develop, the tool may need later refinements. The risk stratification tool will be assessed only once in the study and is not designed to evaluate changes in augmented central pain processing over time. At present, it remains unclear when a change in augmented central pain processing without intervention may occur. The mechanistic understanding and outcome measurement of pain are rapidly advancing, yet remain incomplete. Changes in understanding and the development of new indices of augmented central pain processing may justify adapting the tool we aim to develop. No tool should be regarded as definitive. There is potential for interactions, such as synergy, that may complicate interpretation. It has not been possible to address this without adequately validated and easy-to-apply tools and paradigms to measure nociceptive contributions to pain across diagnostic groups.

## Supplementary material

10.1136/bmjopen-2025-115860 online supplemental file 1

10.1136/bmjopen-2025-115860 online supplemental file 2

10.1136/bmjopen-2025-115860 online supplemental file 3

10.1136/bmjopen-2025-115860 online supplemental file 4

10.1136/bmjopen-2025-115860 online supplemental file 5
