# Supplementary material for: Assessing central nervous system contributions to accelerate musculoskeletal pain diagnosis and treatment (AsCent): protocol for a mixed-method, prospective observational study
Source: BMJ Open. 2026 May 18;16(5):e115860. doi: 10.1136/bmjopen-2025-115860 (PMC13185045; doi:10.1136/bmjopen-2025-115860)
Supplement: online supplemental file 1 [file bmjopen-16-5-s001.pdf]

University of Nottingham
UK | CHINA | MALAYSIA

NHS Sherwood Forest Hospitals NHS Foundation Trust

AsCent Assessing Central Aspects of Pain

CONSENT FORM

(Final Version 3.0 09.01.2025)

Title of study: Assessing Central Aspects of Pain

IRAS Project ID: 343021

Name of Participant:

Name of Researcher:

AsCent ID:

Kindly initial each box as appropriate. INITIAL

1. I confirm that I have read the information sheet dated 29.04.2025 Version 5 for the above study. I have had an opportunity to consider the information, ask questions and have had these answered satisfactorily. ☐

2. I understand that my participation is voluntary and that I am free to withdraw at any time without giving any reason and without my medical care or legal rights being affected. I understand that should I withdraw, then the information collected so far cannot be erased and that this information may still be used in the project analysis. ☐

3. I understand that relevant sections of my medical notes and data collected during the study may be looked at by authorised individuals from the University of Nottingham, the research group, and regulatory authorities or Sherwood Forest NHS Foundation Trust, where it is relevant to my taking part in this research. I give permission for these individuals to have access to my records and to collect, store and analyse and publish information obtained from my participation in this study. I understand that my personal details will be kept confidential. ☐

4. I understand that I would provide a blood sample which researchers may use to measure inflammation and other molecules relevant to arthritis and musculoskeletal pain conditions. I understand that the results of the blood tests and any additional analysis will be anonymised, and my identity will not be linked to these results. ☐

5. I understand that any interviews I participate in will be recorded and that anonymous direct quotes from the interview may be used in the study reports. ☐

6. I understand that anonymised research data may be shared with researchers in universities or organisations, including those in other countries, for research in health and social care. ☐

7. **(OPTIONAL)** I agree that the samples I have given, and the information gathered about me can be stored by the University of Nottingham at the tissue repository for possible use in future studies. I understand that some of these studies may be carried out by researchers other than the current team who ran the first study, including researchers working for commercial companies. Any samples or data used will be anonymised, and I will not be identified in any way. ☐YES ☐NO

8. **(OPTIONAL)** I agree to be sent weekly text messages for 12 weeks, asking me to respond to a question about my pain levels over each past week. ☐YES ☐NO

When complete: 1 for participant, 1 for research site file, 1 to be kept in medical notes.

9. **(OPTIONAL)** I understand that researchers at the University of Nottingham might contact me with further information or invite me to contribute further to their research. I understand that receiving further information or being contacted will not commit me to joining any studies.

☐YES
☐NO

**Name of participant**	**Date**	**Signature**
**Name of person taking consent**	**Date**	**Signature**
**Principal Investigator**	**Date**	**Signature**

When complete: 1 for participant, 1 for research site file, 1 to be kept in medical notes.
