# Supplementary material for: Assessing central nervous system contributions to accelerate musculoskeletal pain diagnosis and treatment (AsCent): protocol for a mixed-method, prospective observational study
Source: BMJ Open. 2026 May 18;16(5):e115860. doi: 10.1136/bmjopen-2025-115860 (PMC13185045; doi:10.1136/bmjopen-2025-115860)
Supplement: online supplemental file 2 [file bmjopen-16-5-s002.pdf]

Assessing Central Aspect of Pain (AsCent) Proposed Interview Topics (HCPs)

Thank you for agreeing to take part in this interview.

To recap the aims of this study, we are...

[Note fire evacuation procedures if in person]

I would just like to remind you that:

- Everything you say as part of this interview will be kept confidential, unless you say anything that indicates you or others may be at harm. If this does happen, we may to report it to someone else, but we will talk to you about this first.
- The information you provide will be anonymised, and any data that is used in the write up of the study results for publication will not be able to directly identify you as an individual.
- The interview will be recorded on Microsoft Teams to help us capture everything that is said. This will help us with our study findings later.
- You do not have to answer all the questions, can take a break if needed, and can withdraw at any point without giving a reason. If you do choose to withdraw, the information and data we already hold about you will be kept and used for the study.

Do you have any questions about the interview at this point? (If yes, answer these. If no, proceed).

We will now begin the interview, and I will start recording in a minute. When the recording starts, I will first ask you to state your name and that you agree to take part in the interview, so we have an additional record.

Start recording

Sections	Main questions	Prompt questions
**Warm up questions**	Check they have viewed the familiarisation video/information provided.<br>Allow them to watch if not already done and provide brief information.<br>Answer any immediate questions.	
**Main question/theme 1**	Can you start by telling me a bit about your role on a day-to-day basis, and how you generally interact with patients with arthritis or chronic back pain?	1. How long have you been in your current role for?<br>2. Are you involved in conducting clinical assessments like the one you saw on the video in your daily practice?<br>a. How often do you conduct these assessments?<br>b. Thoughts about time required in current role to conduct such assessments?<br>3. Can you describe some of the types of pain assessments have you been involved with in the past?<br>a. What have been some of the strengths of these?
**Main question/theme 1**	I would like you to ask you about your thoughts on the usefulness and feasibility of the proposed tool.	1. Can you tell me about your initial thoughts about the tool from the video/information provided?<br>2. Can you see any elements of this tool that may be challenging to conduct for you?<br>a. Or anything challenging for your patients, and why? i.e., barriers<br>3. Which elements make most sense to you in your practice and why?<br>a. i.e., impact, management of current and future practice, changes in clinical reasoning?

		4. What is useful for you or what do you see useful for others?<br>a. What do you like best about this procedure?<br>b. Do you perceive any barriers to using this tool (as a professional/for patients)?<br>5. Please discuss any of these elements you wouldn't want to engage with, and why not?<br>6. What is the most important part of the tool from your perspective if we did cut it down, and why?<br>7. How could you see this fitting in with your current practice/research?<br>a. Do you foresee differences for different patients? (arthritis vs FM vs back pain)<br>8. Who do you think would be best placed to carry out this assessment, e.g., doctor, consultant, nurse, pain management team, etc.?<br>a. What kind of training would be required?<br>b. Who would deliver this, and how would it be delivered (videos, in person).<br>9. How could you see this being useful in your clinical practice/research setting?<br>10. Do you perceive any unintended consequences of using the tool in practice for services/other professionals?<br>11. Please describe how would you see this fitting into your appointments at present?<br>a. Suggestions on efficiency?

**Main question/theme 2**	Please describe the types of pain assessments you have experienced in the past.	1. Tell me how this assessment differs from your past experience(s) of using other similar assessments?<br>2. Please can you describe some of your thoughts on the CAP questionnaire?<br>3. How do you think this tool would be more feasible than others you have used in the past?<br>a. What would you need to be able to use this tool in practice?<br>4. Do you have any further suggestions to improve the bedside assessment tool?
**Main question/theme 3**	Is there anything we haven't asked you that you think is really important to help us understand how to develop and use this tool in clinical practice?	Follow up as appropriate
