# Supplementary material for: Assessing central nervous system contributions to accelerate musculoskeletal pain diagnosis and treatment (AsCent): protocol for a mixed-method, prospective observational study
Source: BMJ Open. 2026 May 18;16(5):e115860. doi: 10.1136/bmjopen-2025-115860 (PMC13185045; doi:10.1136/bmjopen-2025-115860)
Supplement: online supplemental file 3 [file bmjopen-16-5-s003.pdf]

HCP ECONSENT Assessing Central Aspects of Pain Final v3.0 Draft v1.0 17.03.2025

Thank you for your interest in taking part in this research project.

Below you can see the details of the Participant Information Sheet (PIS) for the study entitled: 'Assessing Central Aspect of Pain'.
Please ensure you read this thoroughly, and if you have any questions about the research or your involvement, please contact one of the researcher team listed in the PIS below.
If you are satisfied with the information and would like to provide consent to take part, please ensure you read each of the statements provided on the following page, and if you agree to them, select 'yes' to all mandatory fields (*). You can choose if you wish to receive a copy of the study results, too.
If you do not agree to all the mandatory 'yes' statements, please close the window and do not continue or input any personal data.

Invitation to join the AsCent study:

this study is developing a clinical tool to assess dysfunction in the central nervous system (brain and spinal cord) pain pathways of people with musculoskeletal conditions, such as arthritis, low back pain, or fibromyalgia. We are inviting healthcare professionals (HCPs) and researchers to help us develop the tool to ensure it is feasible and acceptable for use in clinical practice. Before you decide, it is essential to understand why we are doing the study, how we use the information collected, what the study will involve, and the potential benefits, risks, and possible discomforts. Please read this information sheet and either reply to the invitation email or complete the eConsent form if you are interested in participating.

Why is the study being conducted?
Pain persists even in people with well-treated arthritis, low back pain or fibromyalgia. Pathways within the central nervous system sense how we feel pain and can exacerbate pain severity. We refer to this as central sensitisation. Assessments of central sensitisation require extensive training and are expensive. To treat pain effectively, we need to be able to determine central sensitisation, as people with central sensitisation may be unlikely to respond to conventional treatments. Through this study, we aim to develop a tool to measure central sensitisation that can be used during clinical appointments.

Why have I been invited?
We hope that about 27 people will take part. You are being invited to take part because you may meet the below criteria:

- Is aged 18 years or older and
- Work as a healthcare professional or researcher within rheumatology, musculoskeletal health and/or chronic pain management (e.g., physiotherapist, GPs, consultants,

pharmacist)

Taking part is entirely voluntary

It is your decision whether to participate. If you decide to participate, you will receive this information sheet and a copy of the consent form to keep.

What happens if I don't want to carry on?

You are free to withdraw at any time without giving a reason and without affecting your legal or employment rights. You can choose not to take part, now or in the future, by contacting the central AsCent study team on msk-recruitment@nottingham.ac.uk or 0115 823 1676. You will not be disadvantaged in any way. We will tell you if new findings during the study may affect your willingness to participate. If you withdraw from the study for any reason, we will no longer collect information about you or from you, but we will keep the information about you that we have already obtained. We may have already used it in the analysis and may still use it in the final analysis. We will use the minimum personally identifiable information to safeguard your rights.

What are we asking you to do

At a date and time convenient for you, we would like you to: 1. Complete the eConsent form in MS Forms.

2. To familiarise yourself with the proposed tool by watching several short videos (approximately 11 minutes in total) and reading the supporting information. 3. Participate in a short interview conducted on and recorded via Ms Teams, lasting approximately 30 minutes.

Expenses and payments

You will not receive payment or expenses for taking part in this study, as there are no travel requirements since the interviews will be held on MS Teams.

What are the possible benefits and risks of taking part?

Benefits: We cannot promise that the study will help you. The interviews inform the feasibility and acceptability of the proposed clinical tool (simplified clinical quantitative sensory testing and Central Aspects of Pain (CAP) questionnaire) for assessing pain related to central nervous system dysfunction. The information from this study may help us understand the perceptions of those working in the field to ensure the clinical tool is fit for purpose.

Risks: The risks associated with the interview are very low. You may be asked about your previous experiences assessing pain in patients, which may cause some psychological distress. We cannot guarantee that you have not previously had distressing experiences working in the field. You do not have to share any details that you do not wish to.

What happens when the research study stops?

Once the study stops, the study team will cease to contact you, and your involvement with the study will end.

What if there is a problem?

If you are concerned about any aspect of the study, you should speak with the researchers, who will do their best to answer your questions. Their details are at the end of this information sheet. If you are unhappy and wish to complain formally, contact the FMHS Research Ethics Committee Administrator, Faculty Hub, Medicine and Health Sciences, E41, E Floor, Medical School, Queens Medical Centre Campus. Nottingham University Hospitals, Nottingham, NG7 2UH or via Email: FMHS-ResearchEthics@nottingham.ac.uk

In the unlikely event that something goes wrong, and you are harmed during this research, and this is due to someone's negligence, you may have grounds for legal action or compensation against the University of Nottingham. You may have to pay your legal costs.

How will we use information about you?

The University of Nottingham are the sponsor of this study. This means we are responsible for looking after your information and using it properly.

We will need to use information from you for this research project. This information will include your:

- Name
- Contact details
- Age
- Audio-visual recordings of the interviews Via Microsoft Teams
- Occupational role

This information will be used to conduct research and ensure its proper conduct. If you agree, we will keep your contact details to send you the study's findings.

People who do not need to know who you are will not be able to see your name or contact details. Your data will have a code number instead. We will keep all information about you safe and secure. Once we have finished the study, we will keep some of the data so we can check the results. We will write our reports so that no one can work out that you took part in the study.

We may share our research data with research in other Universities or organisations, including those in other countries, for research in health and social care. Sharing research data is important to allow peer scrutiny, re-use (and therefore avoiding duplication of research) and to understand the bigger picture in particular areas of research. Data sharing in this way will be anonymised.

Although what you say to us is confidential, should you disclose anything to use which we feel puts you or anyone else at any risk, we may feel it necessary to report this to the appropriate persons.

What are the choices about how your information is used?

- You can stop being part of the study at any time, without giving a reason, but we will keep information about you that we already have.
- For research to be reliable, we need to manage your records in specific ways. This means that we won't be able to let you see or change the data we hold about you.

Where can you find out more about how your information is used?

You can find out more about how we use your information:

- Reading our privacy statement: https://www.nottingham.ac.uk/utilities/privacy/privacy-information-for-research-participants.aspx
- By asking one of the research team by emailing msk-recruitment@nottingham.ac.uk, or ringing us on 0115 823 1676

- You can contact the Sponsor's Data Protection Officer; dpo@nottingham.ac.uk. Postal address: Data Protection Officer, B16, Lenton Hurst, University of Nottingham

What will happen to the results of this study?

The results of this study may be published in medical literature and made more widely available, for example, through our website (https://www.nottingham.ac.uk/paincentre/). We will not reveal your identity.

Who is organising and funding the study?

The University of Nottingham staff organise and conduct this study. Versus Arthritis and the European Alliance of Associations of Rheumatology funds it through grants paid to the University of Nottingham. The funders do not influence the conduct of the study.

Who has reviewed the study?

An independent Research Ethics Committee reviews all human research to protect your interest. All materials associated with the study have been given a favourable opinion by Yorkshire and Humber - NHS Research Ethics Committee and the Health Research Authority.

Who can I contact if I have any questions about the study?

If you have any further questions about this study or wish to contact the Study Sponsor, please discuss this in the first instance with Dr Stephanie Smith, Chief Investigator. Telephone: 0115 823 1676, Email: msk-recruitment@nottingham.ac.uk

Thank you for taking the time to read this information sheet.

Consent form

Please select 'yes' to all statements if you agree to these and would like to provide consent to participate in this study.

1. I confirm that I have read and understand the information sheet [INSERT PIS VERSION NUMBER AND DATE] for the above study and have had the opportunity to ask questions.*

O Yes

2. I understand that my participation is voluntary and that I am free to withdraw at any time, without giving any reason, and without my legal rights being affected. I understand that should I withdraw then the information collected so far cannot be erased and that this information may still be used in the project analysis.*

O Yes

3. I understand that data collected in the study may be looked at by authorised individuals from the University of Nottingham, the research group and regulatory authorities where it is relevant to my taking part in this study. I give permission for these individuals to have access to these records and to collect, store, analyse and publish information obtained from my participation in this study. I understand that my personal details will be kept confidential.*

O Yes

4. I understand that the interview will be recorded Microsoft Teams and that anonymous direct quotes from the interview may be used in the study reports.*

O Yes

5. I understand that the information collected about me will be used to support other research in the future, and may be shared anonymously with other researchers.*

O Yes

6. I agree to take part in the above study.*

O Yes

7. I would like to receive a copy of the results of this study.*

O Yes

Contact and demographic information

Please provide the following details to help us with study management:

1. Please provide your full name:*

2. Please provide your age:*

3. Please provide brief details about your occupational role:*

4. Please provide your email address so you can be contacted to arrange the interview:*
