# Supplementary material for: Assessing central nervous system contributions to accelerate musculoskeletal pain diagnosis and treatment (AsCent): protocol for a mixed-method, prospective observational study
Source: BMJ Open. 2026 May 18;16(5):e115860. doi: 10.1136/bmjopen-2025-115860 (PMC13185045; doi:10.1136/bmjopen-2025-115860)
Supplement: online supplemental file 4 [file bmjopen-16-5-s004.pdf]

Assessing Central Aspect of Pain (AsCent) Proposed Interview Topics

Thank you for agreeing to take part in this interview.

To recap the aims of this study, we are…

[Note fire evacuation procedures if in person and conducted on a different day to their initial visit]

I would just like to remind you that:

- Everything you say as part of this interview will be kept confidential, unless you say anything that indicates you or others may be at harm. If this does happen, we may to report it to someone else, but we will talk to you about this first.
- The information you provide will be anonymised, and any data that is used in the write up of the study results for publication will not be able to directly identify you as an individual.
- The interview will be recorded on Microsoft Teams to help us capture everything that is said. This will help us with our study findings later.
- You do not have to answer all the questions, can take a break if needed, and can withdraw at any point without giving a reason. If you do choose to withdraw, the information and data we already hold about you will be kept and used for the study.

Do you have any questions about the interview at this point? (If yes, answer these. If no, proceed).

We will now begin the interview, and I will start recording in a minute. When the recording starts, I will first ask you to state your name and that you agree to take part in the interview, so we have an additional record.

Start recording

Sections	Main questions	Prompt questions
**Pre-interview**	How have you been since last we saw you?<br>Check overall recall of study visit process. (only if not after the appointment)	
**Main question/theme 1 (warm-up)**	I would like you to think back to your study visit when we undertook the clinical tool assessment consisting of the 1-page questionnaire and the short pain assessments [first/second half of the visit]. Can you tell me briefly about what happened and how you found it?	1. What is your understanding about what this test offers (in relation to previous experiences)?<br>a. How might you explain the test/tool to somebody else?<br>2. Why do you think it might be important to assess pain in this way? Give examples.<br>3. Which elements were easier or made more sense to you and why?<br>4. Where any of the elements challenging in any way? Why?<br>5. Are there any of these elements you wouldn’t want to engage with, and why not?<br>a. Did you experience any significant side effects to the assessments?<br>b. Were these prohibitive to your day-to-day activities? (only if time between appointments)<br>6. Thinking about the experience, are there things that would have made the experience better or easier for you?<br>7. How would you see this fitting into your appointments?<br>a. Barriers to this?<br>8. How could you see this fitting in with your pain management? - What would that mean for you?<br>a. Barriers to this?<br>9. Who would you anticipate completing this test?

		a. Would you be happy for your clinical care team to carry out this assessment, e.g., doctor, consultant, nurse, pain management team, etc.?<br>10. Could you see this being part of hospital and or GP management?
**Main question/theme 2**	Please describe the types of pain assessments you have experienced in the past.	1. How does this assessment differ from your past experience(s)?<br>a. Was it more pleasant/quicker/easier or not?<br>2. What are your thoughts about the use of the CAP questionnaire as part of the bedside tool?<br>a. Does it add anything?<br>b. Too much to add in?<br>3. Do you think this type of assessment is doable/useful in general practice?<br>4. Do you have any suggestions for what could make the experience of the bedside pain assessment better?
**Main question/theme 3**	Is there anything we haven't asked you that you think is really important to help us understand how to develop and use this tool in clinical practice?	Follow up as appropriate
