# Supplementary material for: Assessing central nervous system contributions to accelerate musculoskeletal pain diagnosis and treatment (AsCent): protocol for a mixed-method, prospective observational study
Source: BMJ Open. 2026 May 18;16(5):e115860. doi: 10.1136/bmjopen-2025-115860 (PMC13185045; doi:10.1136/bmjopen-2025-115860)
Supplement: Supplementary file 6 [file bmjopen-2025-115860.reviewer_comments.pdf]

PEER REVIEW HISTORY

BMJ Open publishes all reviews undertaken for accepted manuscripts. Reviewers are asked to complete a checklist review form and are provided with free text boxes to elaborate on their assessment. These free text comments are reproduced below.

ARTICLE DETAILS

Title (Provisional)

Assessing Central Nervous System Contributions to Accelerate Musculoskeletal Pain Diagnosis and Treatment (AsCent): Protocol for a mixed method, prospective observational study

Authors

Clay, Georgia; Vanhegan, Stevie; Abbott, Caroline; A Pearce, Fiona; Moffatt, Fiona; Bannister, Kirsty; Graven-Nielsen, Thomas; Walsh, David A; Smith, Stephanie

---

VERSION 1 - REVIEW

**Reviewer**	**1**
**Name**	**Keating, Christopher**
**Affiliation**	**Thomas Jefferson University**
**Date**	**25-Mar-2026**
**COI**	**None**

Thank you for the opportunity to review your work. Below are some thoughts and considerations as you look to answer a critical research questions.

Introduction: This section is well supported and clearly identifies the need for precision medicine in its pursuit to find pragmatic ways to identify and guide treatment decisions. Your review is detailed and clearly associated with the RQs and aims.

Methodology: The inclusion criteria rely on self-report for diagnosis of known groups. While this reduces the costs and feasibility of the study recruitment it will alter the sample in the study. These patients may have a stronger identify with their dx and as such have a higher association of psychological distress. Clinical tests or referral may help provide a more generalizable population.

Equally weighing the groups may alter the results as well. You could include this data point as part of the analysis an recruit a more representative group of those with chronic pain and then move into subgroup analysis.

How will the subgroup be determined for the semi-structured interview?

Excellent testing protocol. Very clear.

For the PPT, what if the patient is already in pain?

More details about the HCP and researchers recruited. Where and how are they recruited to provide a clearer picture of what design flaws could be present.

Will the data be made publicly available?

Rasch: was there consideration to the multifactorial nature of pain (latent variable) may result in multiple constructs with a significant residual value when QST and CSI are combined as the screening tool for analysis?

The analysis plan seems appropriate and reasonable to answer the RQs.

Discussion/Conclusion: Very well written and supported through the framework provided in the introduction. I believe this work is very thoughtful and will have a significant impact on risk stratification and instrument development. Well Done!

VERSION 1 - AUTHOR RESPONSE

Reviewer: 1

Thank you for the opportunity to review your work. Below are some thoughts and considerations as you look to answer a critical research questions.

Introduction: This section is well supported and clearly identifies the need for precision medicine in its pursuit to find pragmatic ways to identify and guide treatment decisions. Your review is detailed and clearly associated with the RQs and aims.

Response: Thank you for your kind feedback on our paper.

Methodology: The inclusion criteria rely on self-report for diagnosis of known groups. While this reduces the costs and feasibility of the study recruitment it will alter the sample in the study. These patients may have a stronger identify with their dx and as such have a higher association of psychological distress. Clinical tests or referral may help provide a more generalizable population.

Response: We concur that individuals who self-report their diagnosis may differ from those referred by physicians. As such, we have clarified that participants recruited via primary or secondary care will be identified by a diagnosis done by a physician of one or more MSK conditions, whilst those recruited from the community via research cohorts will self-report. We hope this may also improve the generalisability of the population. Following has been added to the participants section of the methods:

"Individuals recruited from primary or secondary care will have a diagnosis done by a physician of one or more of the above conditions. Individuals recruited from community or research cohorts, will have a self-reported diagnosis."

Equally weighing the groups may alter the results as well. You could include this data point

as part of the analysis an recruit a more representative group of those with chronic pain and then move into subgroup analysis.

Response: We concur that equal weighting of the groups may alter our results. The purpose of purposive sampling is to ensure that less common conditions, such as psoriatic arthritis and ankylosing spondylitis, are not lost in the analysis due to significantly higher prevalence of conditions such as chronic low back pain and osteoarthritis in the population. We anticipate that many participants will have multiple long-term musculoskeletal conditions (e.g., OA + CLBP + FM, or IA + OA), and as such, we think that the total sample will be more representative of the chronic pain population, even when purposefully sampling.

The participants section of the methods now reads: *"Recruitment will ensure the study populations represent the four diagnostic groups, and display age, sex, social and ethnic diversity representative of the study population. Purposive sampling will be used to ensure that less common conditions, such as inflammatory arthritis, have sufficient power to allow proper analysis. Given the prevalence of multiple long-term musculoskeletal conditions, we anticipate that this approach will yield a representative chronic pain population."*

The data analysis section now reads: "Sensitivity analysis will be conducted between diagnostic groups and between matched samples."

How will the subgroup be determined for the semi-structured interview?

More details about the HCP and researchers recruited. Where and how are they recruited to provide a clearer picture of what design flaws could be present.

Response: Thank you for highlighting these important points The following has been added to the nested qualitative interviews section

The nested qualitative interview sections now reads: *"Participants will be purposively sampled intially, with theoretical sampling later to explore emerging themes. Approximately 27 participants, 22 HCPs, and researchers will ensure that perspectives across all conditions – gender, age, ethnicity, social deprivation, job role and career level – are represented (Table 2). Participants will be individuals who have experienced the new tool as part of the study. HCPs and researchers will be recruited via Cwm Taf Morgannwg University Health Board, Sherwood Forest NHS Foundation Trust, Primary Integrated Community Services, social media, word of mouth, and advertising through the British Pain Society. All HCPs and researchers will be provided with an invitation letter, participant information sheet and e-consent (supplementary material 5) to complete if interested"*

Excellent testing protocol. Very clear.

Response: Thank you, appreciated.

For the PPT, what if the patient is already in pain?

Response: All QST measures are conducted regardless of whether someone is in pain. These techniques are well tolerated by individuals with musculoskeletal pain and have been used in >500 individuals with pain previously. Participants are asked to focus on the area where the probe is, particularly when the feeling of pressure changes to pain.

The following sentence has been added to the QST section: *"These measures have previously been well tolerated by individuals with musculoskeletal pain and will be conducted regardless of their pain status".*

Will the data be made publicly available?

Response: Yes, data will be made publicly available via the Advanced Pain Discovery Platform (APDP) Alleviate Data Hub upon publication of the primary findings. The clinicaltrials.gov registration has been updated with this information and the availability statement updated at the end of the manuscript.

Rasch: was there consideration to the multifactorial nature of pain (latent variable) may result in multiple constructs with a significant residual value when QST and CSI are combined as the screening tool for analysis?

Response: Thank you for this important question. It is entirely plausible that multiple constructs with significant residuals may be identified. Further exploration of these constructs would be undertaken to determine whether they measure slightly different things using principal component analysis of the residual values, the eigenvalue criterion, and residual correlations, combined with theoretical underpinning. As such AsCent tool may have multiple linked constructs.

The following has been added to the data analysis section: *"Rasch Measurement Theory, Cronbach's alpha, and confirmatory factor analysis will evaluate internal validity, consistency, and factor structure. If multiple constructs with significant residual values exist, this will be further explored using principal component analysis of residuals, the eigenvalue criterion, residual correlations, and theoretical understanding."*

The analysis plan seems appropriate and reasonable to answer the RQs.

Response: Thank you.

Discussion/Conclusion: Very well written and supported through the framework provided in the introduction. I believe this work is very thoughtful and will have a significant impact on risk stratification and instrument development. Well Done!

Response: Thank you for your kind words about our manuscript.
